# Oxygen Deficiency Modulated La-Doped BaSnO_3_ Films Showing Improved Light Transmittance

**DOI:** 10.3390/ma18081696

**Published:** 2025-04-08

**Authors:** Kai Wu, Wan-Rong Geng, Yin-Lian Zhu, Xiu-Liang Ma

**Affiliations:** 1 Bay Area Center for Electron Microscopy, Songshan Lake Materials Laboratory, Dongguan 523808, China; wukai@sslab.org.cn (K.W.); zhuyinlian@sslab.org.cn (Y.-L.Z.);; 2 Institute of Physics, Chinese Academy of Sciences, Beijing 100190, China; 3 School of Materials Science and Engineering, Hunan University of Science and Technology, Xiangtan 411201, China; 4 Quantum Science Center of Guangdong-Hong Kong-Macau Greater Bay Area, Shenzhen 518000, China

**Keywords:** transparent conductive oxides, LBSO films, aberration-corrected scanning transmission electron microscopies, RP faults, light transmittance

## Abstract

As one of the representative transparent conducting oxides, perovskite-typed La-doped BaSnO_3_ (LBSO) films could be integrated with other perovskite materials to create all-perovskite oxide devices exhibiting exotic physical properties. To overcome the intricate trade-off between conductivity and transmittance in LBSO-based devices, understanding the structural modulating mechanisms of transmittance is definitely crucial. In this paper, the influences of the prevailing Ruddlesden–Popper faults (RP faults) on the transmittance of LBSO films were systematically illuminated, whose density were regulated by the oxygen partial pressures during film growth. High-angle annular dark field (HAADF) STEM and X-ray diffraction (XRD) were employed to characterize the microstructures of the films growing under various oxygen partial pressures and annealing under different oxygen partial pressures. A decrease in RP fault density was observed in the films grown and annealed at high oxygen partial pressures, which displayed improved visible light transmittance. Atomic-scale energy-dispersive spectroscopy (EDS) and electron energy-loss spectroscopy (EELS) analyses revealed the different electronic structure at RP faults compared with the bulk material, including the double concentration of La and increased M5/M4 white line ratio, which is modulative by the oxygen deficiency in LBSO film. It is revealed that the RP defaults in LBSO films annealed at low oxygen pressures displayed larger changes in electronic structure compared with the counterparts with low oxygen deficiency. This work suggests that the oxygen deficiency in LSBO films plays a crucial role in changing the density of RP faults and their electronic structures, thereby regulating the transmittance of LBSO films, which would provide guidance for fabricating high-performance LBSO films.

## 1. Introduction

Transparent conductive oxides have garnered great attention from researchers due to their outstanding conductivity and transparency, which hold promising applications in organic light-emitting diodes, solar cells, energy conversion, flat-panel displays, and sensors [1,2,3]. Previous studies mainly focused on binary transparent conductive film systems, such as In_2_O_3_, SnO_2_, and ZnO [4,5,6], which encountered evident limitations of raw material constraints. Additionally, the light transmission range, especially in the ultraviolet spectrum, is insufficient, limiting the improvement of light conversion efficiency [7]. Recently, some ternary materials have received great attention as alternative photoelectric materials, such as spinel-structured MgAl_2_O_4_ and MgGa_2_O_4_ [8,9], pseudobrookite-structured FeTi_2_O_5_ [10], and perovskite-structured stannate (MSnO_3_; M = Ba or Sr) [11,12]. Among them, perovskite-structured La-doped perovskite BaSnO_3_ (LBSO) has demonstrated high carrier mobility and wide bandwidth, attracting widespread interest [13,14,15]. In bulk single-crystal BaSnO_3_ with slight La doping, carrier mobility reaches up to ~320 cm^2^ V^−1^ S^−1^, the highest value among room-temperature TCOs, and its electrical performance exhibits remarkable thermal stability, with conductivity variation in air at 530 °C being less than 2% [16].

Considering the practical applications, the LBSO is expected to exist as the film state [17,18,19]. It is worthwhile to note that the carrier concentration of epitaxially grown LBSO thin films could reach bulk levels [14]. However, the mobility of these films is substantially lower than that of single-crystal bulk due to the presence of a considerable number of dislocation and stacking fault defects in these films, which generally arise from the growth conditions or the large lattice mismatch with the substrate used during epitaxial growth [20,21]. Although previous studies concluded that growth process parameters significantly influence the carrier concentration and mobility of LBSO films [22,23,24,25,26], investigations on the effects of different process parameters remain largely at the macroscopic level, focusing predominantly on performance comparisons. However, the atomic-resolved structural mechanisms still remain elusive. Therefore, analyzing the microstructure of thin films under different process parameters at the microscopic scale could provide significant insights into material preparation and performance optimization.

Aberration-corrected transmission electron microscopy (ACTEM) is a critical technique for characterizing defects such as dislocations and stacking faults at the atomic scale, providing insights into their structure, chemical compositions, and electronic states [27,28,29,30]. Paik et al. performed atomic-scale characterization of mixed extended dislocations in BaSnO_3_ thin films using HAADF-STEM. Similarly [31], Yun et al. utilized HAADF-STEM to conduct detailed characterization of misfit dislocations in BaSnO_3_ thin films grown on different substrates, discovering that these dislocations induce both in-plane and out-of-plane rotations of the BaSnO_3_ lattice, thereby affecting the quality of the epitaxial films. Additionally, they identified unique structures such as one-dimensional metallic line defects within the BaSnO_3_ thin films [32,33]. Wang et al. systematically characterized the atomic structure and chemical composition of RP stacking faults in LBSO films using HAADF-STEM and EELS, though they did not correlate these findings with process parameters and performance [34]. It is evident that HAADF-STEM and EELS techniques play a significant role in defect characterization of epitaxial film. Studying the influence of various growth parameters on epitaxial film defects using ACTEM is essential for optimizing their performance.

During the growth of oxide thin films, deposition temperature and oxygen partial pressure serve as two critical parameters. Elevated temperatures facilitate film crystallization and enhance epitaxial growth, while excessive thermal energy may induce elemental volatilization and compromise film densification. The oxygen partial pressure regulates oxygen vacancy concentration, thereby modulating functional properties such as ferroelectric, optoelectronic, and ferromagnetic characteristics [35]. Notably, oxygen partial pressure plays a critical role in tailoring the functional properties of LBSO thin films through vacancy-mediated electronic structure engineering.

In our study, advanced aberration-corrected transmission electron microscopy (TEM) and X-ray diffraction (XRD) were employed to investigate the microstructure of LBSO films prepared under different growth and annealing oxygen partial pressures (PO_2_). High-density Ruddlesden–Popper (RP) faults were observed, and the low PO_2_ films exhibit higher RP fault density accompanying the decreased light transmittance. Atomic-level spectrometry and electron energy loss spectroscopy (EELS) were utilized to analyze the atomic structure and electronic structure of the RP faults. It found that the enrichment of La in RP faults accompanied changes in chemical valence states. The above results revealed the modulated mechanisms that oxygen deficiency affects the density and electronic structure of RP faults in La-doped BaSnO_3_ epitaxial film and thereby the light transmittance property, which could give a further guidance for the fabrication of high-quality La-doped BaSnO_3_ epitaxial films.

## 2. Materials and Methods

A series of epitaxial LBSO thin films were grown on KTaO_3_ (KTO) substrates using pulsed laser deposition (PLD). The process employed a Coherent ComPex PRO 201 F KrF excimer laser with a wavelength of 248 nm. The lattice constant of the KTO matrix is 3.990 Å, while that of LBSO is 4.116 Å. The lattice mismatch between them is calculated as 3.07%. The KTO (001) substrates, which are commercially available, were prepared by heating to 850 °C for 10 min prior to deposition in order to clean their surfaces. For the deposition of LBSO films, a target consisting of BSO doped with 0.05 La was used. This target underwent a pre-sputtering process for 5 min to ensure surface cleanliness. During the growth of the LBSO layers, the laser energy was set at 1.5 J cm^−2^, with a repetition rate of 4 Hz, a substrate temperature maintained at 850 °C, and an oxygen partial pressure of either 90 or 70 mTorr. Following deposition, the films were subjected to annealing at 850 °C under an oxygen partial pressure of 200 Torr or near vacuum (10^−5^ Torr) for 20 min. Subsequently, they were cooled gradually to room temperature at a rate of 5 °C per minute.

High-resolution XRD and reciprocal space mappings measurements were carried out in out-of-plane modes, using Cu Kα radiation in an X-ray diffractometer (XRD, X’Pert3 MRD, Malvern Panalytical, Almelo, The Netherlands). Ultraviolet–visible transparent spectroscopy was performed using a UV-Visible Spectrophotometer (UV-Vis, V-770, JASCO Corporation, Tokyo, Japan).

HAADF-STEM imaging has emerged as a powerful technique for investigating crystal structures at sub-angstrom resolution. Although the HAADF-STEM imaging mechanism involves complex physics, the observed atomic contrast predominantly follows a Z^n^ dependence (where Z represents atomic number, with *n* = 1.7 approaching the asymptotic Rutherford scattering limit Z^1.7^), enabling straightforward image interpretation [36]. EELS is an analytical technique that interrogates the physical and chemical properties of materials through electron–matter interactions. Operating on the principle of inelastic scattering events, EELS precisely quantifies energy losses incurred by incident electrons traversing the specimen, enabling simultaneous determination of elemental composition, chemical bonding configurations, and electronic structure characteristics with atomic-scale spatial resolution [37,38]. Cross-sectional samples for STEM observation were fabricated following a conventional procedure, which included slicing, gluing, grinding, dimpling, and ultimately ion milling. Ion milling was performed using a Gatan 695 PIPS system. Initially, an ion milling process was carried out at a voltage of 4.5 kV and an incidence angle of 7°. Subsequently, the angles were progressively decreased to 5°, and the final voltage was lowered to 0.5 kV to remove any amorphous layers on the sample surfaces. A comparable technique was employed for preparing planar-view TEM samples, where ion milling was conducted exclusively from the substrate side. High-angle annular dark field (HAADF) STEM images were captured using an aberration-corrected (scanning) transmission electron microscope (Spectra 300, Thermo Fisher Scientific, Waltham, MA, USA, equipped with dual aberration (Cs) correctors provided by CEOS and a monochromator, operating at 300 kV). Electron energy-loss spectroscopy (EELS) data were collected using the Spectra 300 microscope integrated with a Gatan 1069 GIF system and K3 camera.

The quantitative assessment of the EDS data was carried out using FEI’s Velox software, version 3.12.1. The integrated spectra underwent correction via an empirical background adjustment method, and quantification was performed utilizing the semi-empirical Schreiber–Wims ionization cross-section model, a method well-suited for metal oxides.

For strain analysis, the study relied on GPA [39], with the specific computations executed through the Strain++ software (https://jjppeters.github.io/Strainpp/).

## 3. Results

### 3.1. XRD and RSM

We have fabricated LBSO films with three different deposition parameters, which were different growth oxygen partial pressures (i.e., 70 mTorr/90 mTorr) and different annealed oxygen partial pressures (i.e., 200 Torr/0 Torr). Specific deposition parameters were 90 mT-200 T, 70 mT-200 T, and 90 mT-0 T, so these three films were named 90-200, 70-200, and 90-0.

**Figure 1:**
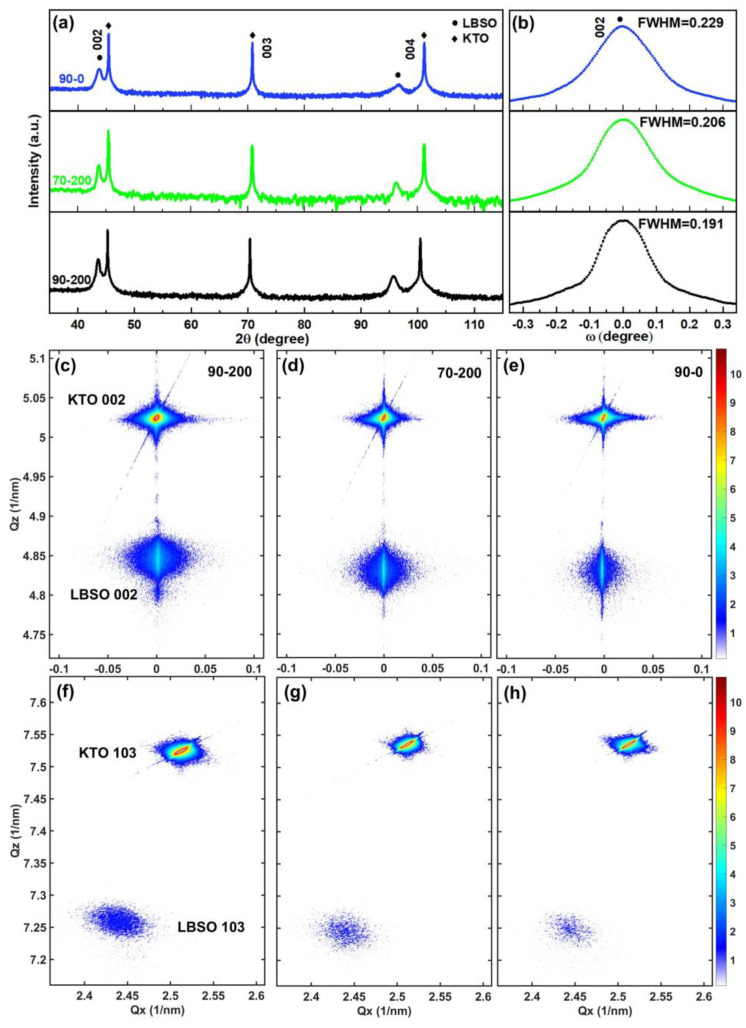
High-resolution XRD patterns of LBSO film grown (001) KTO substrate measured in triple-axis geometry. (**a**) θ-2θ scan; (**b**) rocking curves of the 002 LBSO film peak; (**c**–**e**) reciprocal space map around the 002 peaks; (**f**–**h**) reciprocal space map around the 103 peaks. The film peaks are labeled with black dots.

Figure 1a displays the high-resolution XRD patterns of LBSO films deposited under various conditions. The patterns show only the (002) and (004) peaks of LBSO, which align with the substrate KTO. This indicates that all films exhibit excellent (001) epitaxial growth perpendicular to the substrate, maintaining a pure perovskite structure without any impurity phases. Additionally, the positions of the (004) peaks shift by approximately 0.4° under varying oxygen partial pressures, suggesting an expansion in the out-of-plane lattice parameter. Furthermore, the (00l) peaks of 90-0 LBSO are wider than the other two patterns.

The XRD rocking curves (Omega scans) for the LBSO 002 peaks were obtained using a triple-axis configuration, with the results presented in Figure 1b. The full widths at half maximum (FWHMs) were measured as 0.229°, 0.206°, and 0.191° for the 90-0, 70-200, and 90-200 LBSO films, respectively. This relatively broadened rocking curve can be attributed to structural relaxation caused by the introduction of dislocations and stacking faults during the growth of highly mismatched films (~3.4%) on KTO substrates. Comparing the rocking curves of the three films, it can be concluded that the 90-0 LBSO film has the widest FWHM of 0.229°. The wider half-height width indicates that the 90-0 film has a higher defect density than the other two kinds of films; this can be attributed to the vacuum annealing process of the 90-0 film.

To further confirm the epitaxial strain state of LBSO/KTO films, reciprocal space mapping (RSM) measurements were performed on the LBSO (002) and (103) peaks, as shown in Figure 1c–h. In the LBSO (002) peak in Figure 1c–e, a clear LBSO peak together with satellite peaks had been measured, which indicated a good epitaxial relation between the LBSO film and KTO substrate. The corresponding out-of-plane lattice parameters of the c axis were 4.14 Å, 4.14 Å, and 4.14 Å, respectively. In the (002) RSM images of the three types of LBSO thin films, the LBSO peaks are elongated along the [001] direction. This elongation of diffraction points in reciprocal space suggests the potential presence of numerous planar defects within the films, aligned perpendicular to the film/substrate interface. The interference fringes are periodic intensity oscillation phenomena caused by crystal dynamical diffraction effects in X-ray or electron diffraction [40]. These fringes clearly visible along the central axis of the (002) RSM images indicate a good coherent epitaxial relationship for all three types of films. Additionally, the distinct diffuse scattering signal near the (002) diffraction peak is attributed to the interface misfit dislocations.

In the LBSO (103) peak in Figure 1f–h, the corresponding out-of-plane lattice constants of this LBSO film were calculated to be 4.14 Å, 4.14 Å, and 4.13 Å, and the in-plane lattice constants were calculated to be 4.09 Å, 4.09 Å, and 4.10 Å for 90-200, 70-200, and 90-0, as shown in Table 1. The smaller in-plane lattice constants indicate that the LBSO films were subjected to compressive strain in plane. The in-plane lattice constant of the films grown under the near-vacuum annealing process is slightly higher than that of the other two films annealed under 200 Torr oxygen pressure, which may owe to the defects such as oxygen vacancies and layer faults generated by the low oxygen pressure condition [41,42].

### 3.2. STEM

#### 3.2.1. Cross-Section HAADF-STEM Images

**Figure 2:**
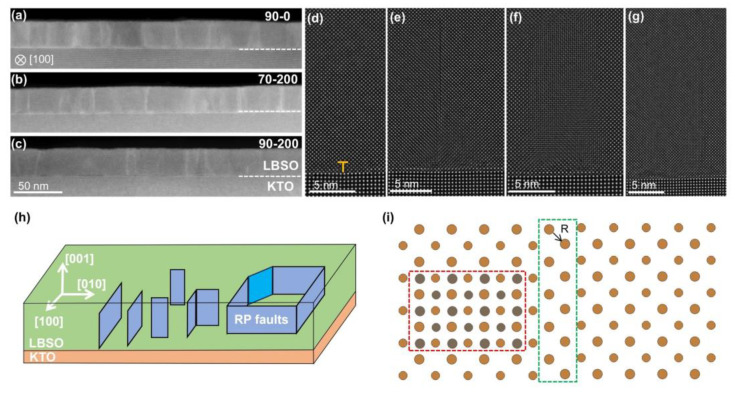
Cross-section HAADF images along the [100] axes of LBSO film: (**a**–**c**) cross-sectional HAADF-STEM images of three films, 90-0, 70-200, and 90-200; (**d**) LBSO/KTO interfacial misfit dislocations; (**e**) (010) RP faults projected in [100] direction; (**f**) (100) RP fault plane projected in the [100] direction; (**g**) (010) and (100) RP fault plane coupled projected in the [100] direction; (**h**) RP faults existed in the film in the way; (**i**) atomic distribution map of (010) in red dotted boxes and (100) RP fault plane projected in the [100] direction in green dotted boxes.

We investigated the defect structure of the LBSO film with different deposition parameters, the same sample whose XRD pattern is shown in Figure 1, by scanning transmission electron microscope (STEM). The cross-sectional HAADF-STEM images of these three films of the entire film thickness are shown in Figure 2.

The microstructure of LBSO/KTO interfaces is shown in Figure 2 a to c, respectively. The film thickness is approximately 25 nm. The magnified image on the right shows a relatively smooth and clean interface with no misoriented grain growth, maintaining good coherence with the substrate. Defects extending perpendicularly to the film/substrate interface, with gray-white contrast, are observed in all three types of films. Additionally, misfit dislocations, which relieve mismatch strain, are present at the interface as shown in Figure 2d. The gray-white contrast is caused by planar defects traversing the film growth, commonly referred to as Ruddlesden–Popper (RP) faults, with a displacement vector of 1/2a<111>. In the [100] projection, these faults exhibit a displacement vector of 1/2a<011>, as illustrated in Figure 2i.

In the LBSO/KTO heteroepitaxial films, the in-plane a and b axes of the LBSO thin films are subjected to equivalent compressive strain from the substrate, resulting in RP fault planes existing on both the (100) and (010) planes. As shown in Figure 2e, the (010) RP fault plane observed in the [100] projection appears as a line on the substrate and exhibits significant lattice expansion, with atomic arrangements indicated by the green rectangular frame in Figure 2i. The (100) RP fault plane observed in the [100] projection presents a densely packed region of atoms without lattice expansion, as depicted in Figure 2f. This occurrence is due to the RP faults causing a 1/2a<101> displacement of the crystal before and after the fault plane. In the [100] projection, the overlapping planes create the illusion of dense packing, as shown by the red rectangular frame in Figure 2i. Besides isolated (100) and (010) RP fault planes, coupled (100) and (010) fault planes are also observed, as shown in Figure 2g. In summary, the presence of RP fault planes in the films exists in several forms: standalone (010) and (100) RP fault planes, coupled (010) and (100) fault planes, and regions enclosed by RP faults, as illustrated in Figure 2h. Notably, within the annular RP fault region, a 1/2a<110> stacking fault is identified, which is relatively rare in LBSO films. Detailed examination of this fault will be presented in the in-plane STEM-HAADF analysis.

Comparing the cross-sectional HAADF images of the three processes, we find that the films exhibit different RP fault densities. The 90-0 film shows the highest fault density, possibly due to the increased RP faults resulting from oxygen deficiency during near-vacuum annealing. To provide a clearer comparison of RP fault density and atomic structure, planar HAADF-STEM and EDS characterizations were performed on each film.

#### 3.2.2. In-Plane HAADF-STEM Images

**Figure 3:**
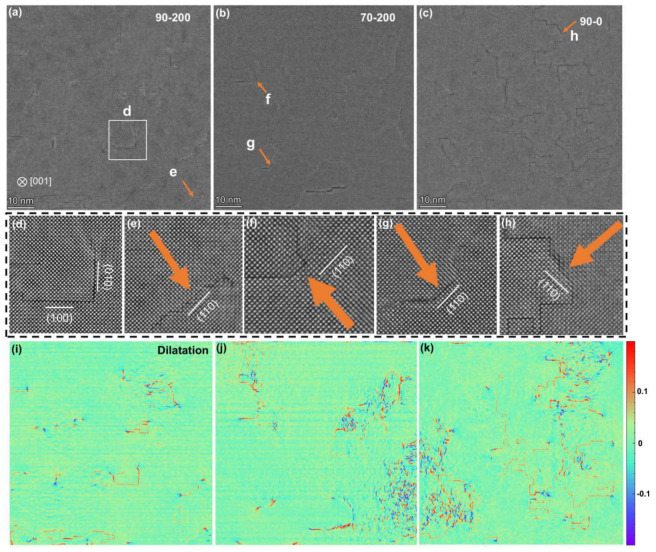
In-plane HAADF images of LBSO films: (**a**–**c**) in-plane HAADF-STEM images of three films of 90-0, 70-200, and 90-200; (**d**–**h**) is the corresponding enlarged images in Figure (**a**–**c**); (**i**–**k**) corresponding strain analysis plots with color bar.

**Figure 4:**
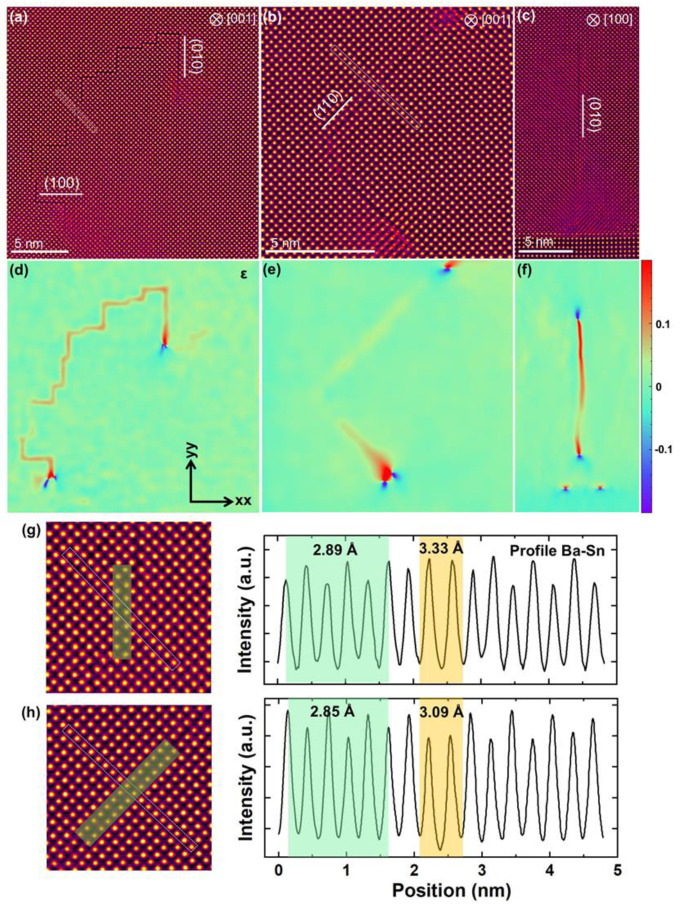
Stacking faults strain analysis: (**a**) {100} RP fault plane projected in the [001] direction; (**b**) 1/2 a <110> type SFs projected in the [001] direction; (**c**) (010) RP fault plane projected in the [100] direction; (**d**–**f**) strain distributions corresponding to Figure (**a**–**c**); (**g**,**h**) lattice profile along the [$\overset{-}{1}$10] direction across the RP faults and 1/2a<110> stacking faults, which is marked by a yellow shaded box in left-hand HAADF image.

Figure 3 shows in-plane HAADF images of the three LBSO films, projected along the [001] direction. In this projection, (010) and (100) fault planes appear as line defects with significant lattice expansion, as shown in Figure 3i–k. Strain analysis using GPA allows us to visualize the distribution of RP faults in each film. Comparing the GPA images reveals that all films contain some RP faults, with these faults being more dispersed in the 90-200 and 70-200 films, while being densely packed in the 90-0 film. This is consistent with results from XRD and cross-sectional HAADF images. As indicated by the yellow arrows in Figure 3e–h, a few 1/2 a <110> stacking faults (SFs) are also present in the films. Additionally, RP faults are always accompanied by compressive strain at the edges and tensile strain across the fault region. Detailed strain analyses of RP and 1/2 a <110> SFs were conducted, as shown in Figure 4.

In this section, the straightforward evolution of lattice parameters was investigated employing the geometry phase analysis method. Figure 4a,b illustrate in-plane HAADF images of two types of SF, and Figure 4c displays a cross-section HAADF image of RP fault. The corresponding relative lattice strains (ɛ*_xx_* + ɛ*_yy_*) are plotted in Figure 4d–f, respectively. The ɛ*_xx_* and ɛ*_yy_* represent relative values that indicate local lattice displacements from the reference LBSO lattice in both horizontal and vertical directions. A positive or negative value of ɛ signifies that the measured local lattice parameters are either larger or smaller than the reference value, respectively. It is evident that the SFs display significant tensile lattice strains perpendicular to their orientation, videlicet lattice expansion. The compressive strain with blue contrast can be observed at both ends of two types of SFs in Figure 4d–f. The presence of misfit dislocations accommodating compressive strain at the LBSO/KTO interface is evident in the cross-sectional GPA images. Above the interface, a strain-free region is observed, followed by a distinct RP fault. This indicates that RP faults are not directly generated by compressive stress at the interface but rather form within the defect-free LBSO film region. This phenomenon occurs as RP faults involve shear strain, which cannot nucleate directly at the interface [43]. Furthermore, a quantitative analysis of lattice expansion at both types of SFs is presented in Figure 4g–h. For ease of comparison, atomic planes along the [110] direction at the center of both fault types were selected, as highlighted by the white rectangular boxes in Figure 4.

In Figure 4g, the HAADF image shows the RP fault on the left, while the right side illustrates the atomic profile along the (110) plane. The interatomic column spacing away from the fault region is measured at 2.89 Å, whereas within the fault region, it increases to 3.33 Å. The atomic column intensity within the fault is higher than that on either side, consistent with the presence of Ba (La) atoms, which possess higher atomic numbers. Conversely, Figure 4h depicts a (110) fault on the left and the corresponding (110) plane atomic profile on the right. Here, the interatomic column spacing away from the fault region is 2.85 Å, expanding to 3.09 Å within the fault region. However, the atomic column intensity within the fault is lower, indicating that the atoms present are Sn, which have lower atomic numbers compared to Ba (La).

The comparison of the two fault regions reveals that the atomic column spacing at the RP fault is greater than at the 1/2 a <110> SFs, indicating a larger tensile strain at the RP fault. From a strain perspective, the 1/2 a <110> SF is more stable than the RP fault. Yet, experimental observations show an abundance of RP faults and a relative scarcity of 1/2 a <110> SFs. This might be attributed to the lower surface energy of (100) planes compared to (110) planes in the perovskite structure. Moreover, a distinctive difference in the atomic structures at the two fault types is demonstrated, and a detailed characterization of these atomic structures will follow.

### 3.3. EDS

**Figure 5:**
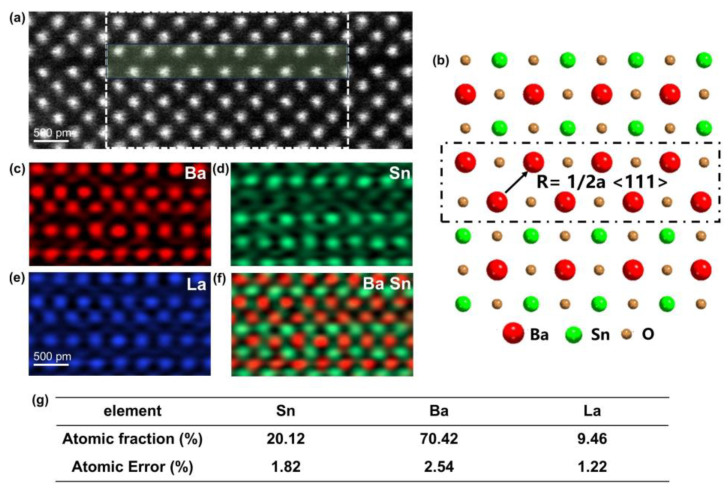
(**a**) An atomic-resolution HAADF-STEM image showing the RP fault. (**b**) An atomic model of the RP fault, with Ba, Sn, and O depicted by red, green, and yellow spheres, respectively. (**c**–**e**) Atomic resolved EDS elemental mapping of Ba, Sn, La. (**f**) The integrated mapping of Ba and Sn. (**g**) The quantitative EDS results of Sn, Ba, and La in the light green rectangular box in (**a**).

The chemical characteristics of stacking faults (SFs) are further analyzed using atomic-resolution EDXS mapping combined with spectrum profile quantification. Figure 5a presents the atomic-resolution HAADF image of the (010) RP fault, viewed along the [001] zone axis. In complex materials, SFs can be categorized into two types: conservative and non-conservative SFs. This classification depends on the relationship between the lattice periodicity in the normal direction of the SF and the magnitude of translational displacement [44]. For conservative SFs, the stoichiometric ratio matches that of the perfect crystal regions, while for non-conservative SFs, an excess of specific elements is typically observed. The elemental composition of these two types of SFs can be clarified through element mapping. In the case of the RP fault, as the displacement vector is not an integral multiple of the periodic vector of the (100) plane, an excess of elements is anticipated [45]. The white dashed rectangles indicate EDS imaging regions. Atomic-level EDS imaging reveals the distribution of Ba, Sn, and La elements within the RP fault region, as depicted in Figure 5c–e. Across the entire region, Ba and La atoms are located in the same atomic columns, suggesting that the A-site-disordered LBSO has been successfully obtained. In the RP fault region, Ba or La segregation forms NaCl-type BaO or LaO structures, unlike the alternating Sn and Ba (La) atomic planes in the perfect region’s ABO_3_ structure. An atomic model of the RP fault was constructed based on these element distributions, as shown in Figure 5b. The RP fault involves a pair of Ba or La atomic planes with a displacement vector R = 1/2<111> a, forming an anti-phase boundary. Quantitative analysis of atomic ratios in RP fault regions was conducted. The family for quantification includes the L lines of Ba, Sn, and La elements, and the ionization cross-section model used is the Schreiber–Wims model, which can give better accuracy for transition metals and metal-oxide. Figure 5g provides quantitative results from the light green area in Figure 5a, showing Sn, Ba, and La atomic proportions of ~20%, ~70%, and ~9.5%, respectively, excluding oxygen due to inaccuracies. It can be seen that Sn atoms still occupy a certain proportion at the fault of the RP layer, which is due to the planar TEM sample. Because the RP layer fault is a kind of plane shear strain, it will not be directly generated from the substrate, but will be generated on the perfect epitaxial film layer of a certain thickness, so about 20% of Sn atoms will appear in the RP layer fault region [43]. In order to compare the proportion of Ba and La elements, we also carried out quantitative calculations in the perfect block region, and the results are shown in Supplemental Material Figure S1, which revealed a Ba atomic ratio of 53.8% and La of 2.6%, closely matching the target doping level of La_0_._05_/Ba_0_._95_. In RP fault regions, La concentration reached 9.46%, and Ba concentration was 70.42%, significantly higher than the bulk La/Ba ratio, indicating La segregation at RP faults. To enhance the concentration of charge carriers in LBSO films, doped La ions are +3 valence. LaO layer of NaCl structure formed in RP region, where La ions should be +2 valence, may contribute to increased RP fault density in near-vacuum annealed films. Detailed valence state information is presented in subsequent EELS results.

**Figure 6:**
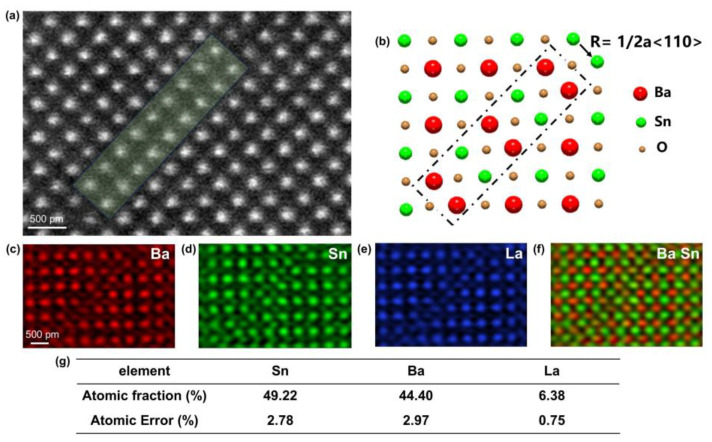
(**a**) An atomic-resolution HAADF-STEM image of the 1/2a<110> stacking fault. (**b**) An atomic model illustrating the stacking fault, with Ba, Sn, and O represented by red, green, and yellow spheres, respectively. (**c**–**e**) Atomic resolved EDS elemental mapping of Ba, Sn, and La. (**f**) The integrated mapping of Ba and Sn. (**g**) The quantitative EDS results of Sn, Ba, and La in the light green rectangular box in (**a**).

Then, we also performed atomic resolved EDXS mapping of the 1/2 a<110>-type SF. Its atomic structure, elemental distribution, and atomic quantization structure are shown in Figure 6. Figure 6a shows the atomic resolved HAADF image of this SF, whose stacking fault plane is parallel to (110). The distribution of Ba, Sn, and La elements is shown in Figure 6c–e, where Ba and La elements still occupy the same position, according to the fact that the Ba (La) and Sn elements are biased on both sides of the SF. We constructed the atomic structure of the SF based on the atomic resolved EDXS mapping of each element, as shown in Figure 6b. It can be seen that the elemental distribution of the 1/2 a<110>-type SF is very different from that of the RP fault. The Ba and Sn elements are arranged alternately along the direction of the layer fault [110], and the bias aggregation of the Ba (La) elements at the SF region does not occur as in the RP fault region. From Figure 6f, it is evident that the 1/2 a<110>-type SF maintains the Ba:Sn = 1:1 stoichiometric ratio of ABO_3_, and thus, this SF is a conserved type of SF. It has been reported in the literature that 1/2 a<110>-type SFs are formed by full dislocation decomposition [34]. Similarly, we quantitatively analyzed the elemental distribution in the 1/2 a<110>-type SF, and the quantitative region is the light green SF region in Figure 6a. The quantitative results are shown in Figure 6g, with the ratio of Sn atoms 49.22%, the ratio of Ba atoms 44.4%, and the ratio of La atoms 6.38%, and the ratio of Sn atoms to Ba + La atoms is close to 1:1, which is consistent with the stoichiometric ratio. As a comparison, we also performed quantization analysis in the fault-free block region of this figure, and the results are shown in Figure S2, with 48.3% of Sn atoms, 49.13% of Ba atoms, and 2.57% of La atoms. Comparative results reveal that the percentage of La atoms at the 1/2a<110>-type SF has significantly increased relative to the fault-free block region, which means that the La elements at the 1/2 a<110>-type SF also undergo the same phenomenon of bias aggregation.

We found that the concentration of La is higher than that in the perfect block region in the two faults region, and the local structure of the RP faults has been converted from an ABO_3_ perovskite structure to an (AO)_2_ rock salt structure. The element La is a valence-changing element, and the valence has a semi-stable +2 valence besides the stable +3 valence [46,47]. The bias aggregation of La in the fault plane may cause a change in valence. In the following, we analyzed the valence states of La in the RP faults region and La in the perfect block region by EELS.

### 3.4. EELS

**Figure 7:**
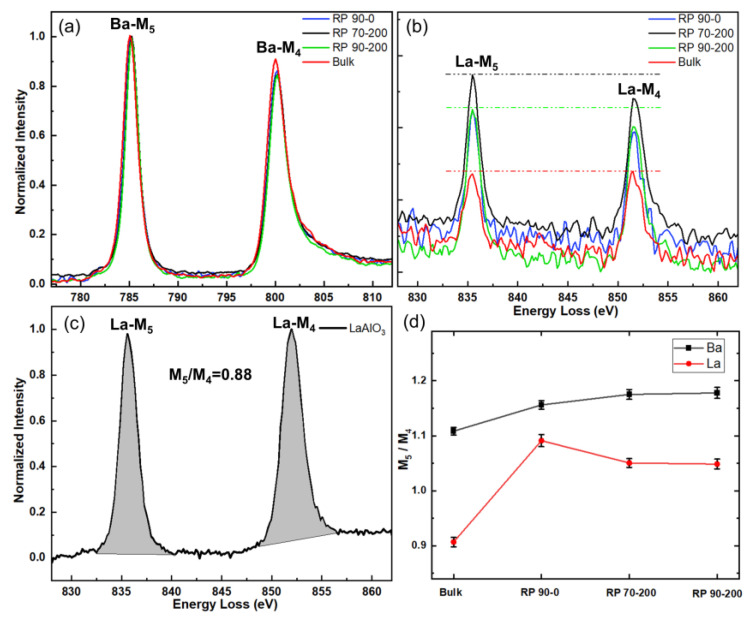
EELS spectra images. (**a**,**b**) Ba and La M spectra acquired from RP faults region and perfect bulk region far from RP faults of LBSO films with different deposited parameters. (**c**) La M spectra acquired from perfect bulk region in LaAlO_3_, white intensity ratio M_5_/M_4_ is 0.88 calculated from the shadow area of M_5_ and M_4_ peaks. (**d**) The calculated Ba and La M_5_/M_4_ white line intensity ratio with error bar from (**a**) and (**b**), respectively.

Figure 7a,b demonstrate the comparison of EELS peaks of Ba and La elements M5 and M4 acquired at the RP faults region of the three films, as well as at the perfect region without defects. The thickness of the acquisition area and the detailed parameters of the acquisition are shown in Figure S3, and the intensity of the Ba-M_5_ peaks was used for normalization. Comparison of the EELS peaks of Ba in the four regions reveals that the peaks in the perfect region are slightly shifted forward, and the intensity of the M4 peak is higher than that in the RP fault region. This chemical shift and the relative change in the peak intensity indicate that there is a change in chemical valence state of Ba in the RP faults region relative to Ba in the perfect region. Since the stable chemical valence state of Ba is only +2 valence, this change in valence state may be due to oxygen vacancies. As illustrated in Figure 7b, by comparing the EELS peak intensities of La in the four regions, it is evident that the peak intensity in the bulk region is considerably lower than that in the RP fault region. This suggests that the concentration of La elements in the bulk region is less than that in the RP fault region, thereby validating the quantitative results obtained from the previous EDS analysis. In addition, the M_4_ and M_5_ intensities of La elements in the bulk region are almost equal, and the M_5_ peak intensity of La elements in the RP faults region of the three films are higher than those of the M_4_ peaks, which also indicates a change in the chemical valence state of the La element. The stable chemical valence state of the La element is +3, and metastable chemical valence state +2 also exists. Generally, the doped La element is usually considered to be a stable +3 valence [24].

The M_5_ and M_4_ peaks of La originate from the excitation of electrons from the 3d_3/2_ and 3d_5/2_ core levels to the unoccupied 5d or 4f band. This phenomenon is utilized to determine the valence state of rare earth elements through the white line ratio or chemical shift [48]. We quantified the white line ratio, as shown in Figure 7d. For comparing, we calculated the white line ratio of La^3+^ references from the LaAlO_3_ film, as shown in Figure 7c. The elemental valence of La in LaAlO_3_ is +3 and the calculated white line ratio M_5_/M_4_ is about 0.88. We observe that the M_5_/M_4_ ratio in the RP faults region increases compared with that in the fault-free region, suggesting a change in valence state of the La element. In the fault-free region far from the fault planes, the valence of La is closed to +3, as expected. According to the literature [49], the peak intensity of M_5_ of the La^2+^ ion is slightly higher than that of M_4_, and the peak intensity of M_5_ of the La^3+^ ion is close to that of M_4_. Therefore, we can judge that La ion in the RP layer fault region is +2 valence. In the fault region, some of the La ions become metastable +2 valence, which leads to an increase in the value of the white line ratio. The La M_5_/M_4_ intensity ratio in the RP fault of 90-0 film is higher than that in the RP fault of 90-200 and 70-200 film, indicating a lower average valence of La around the RP fault of 90-0 film. Further, the Ba M_5_/M_4_ intensity ratio in the RP fault region is a little higher than that in bulk region, indicating a lower average valence of Ba around the RP fault region.

### 3.5. Optical Transmission

**Figure 8:**
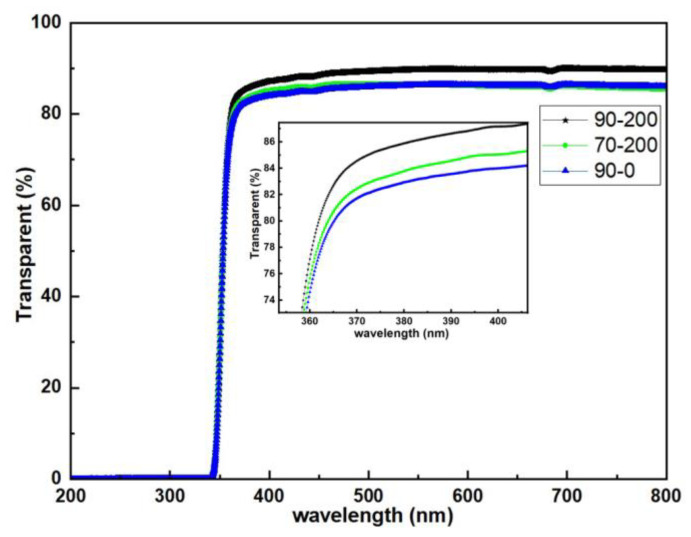
Optical transmission of LBSO films with different deposited parameters.

Finally, we characterized the visible light transmittance of the three films, and the results are shown in Figure 8. The transmittance of the three films is good, which is more than 80%. From the magnified comparison graph in Figure 8, we can see that the 90-200 film has the highest visible light transmittance, followed by the 70-200 film, and the lowest being the 90-0 film. It has been reported that strain gradient leads to enhanced visible light absorption [50]. From the XRD results, cross-sectional HAADF images and planar HAADF images, it is known that the defect density is the highest in the 90-0 film. The higher defect density leads to more distortions in the lattice, inhomogeneity of the strain field, and enhancement of the visible light absorption, which reduces the visible light transmittance.

## 4. Discussion

Combined with the valence changes of La ions in the RP faults region, we can discuss the effect of different deposition parameters on the RP fault density of the films. The largest RP fault density among the three films is 90-0, which has the same process parameters as 90-200 except for the annealed oxygen partial pressure, which means that more RP defects are produced under the near-vacuum annealing process. From the EDS quantitative results and EELS valence analysis, it can be known that there is an enrichment of La ions in RP faults region, and at the same time, the valence of some of the La ions reduced from +3 to +2 valence. In the annealing process, oxygen atoms in the LBSO film will be missing to form oxygen vacancies, and the film in the state of oxygen deficiency (oxygen vacancies) part of the La^3+^ ions is reduced to metastable La^2+^ ions, while the energy provided by annealing makes the La^2+^ ions migrate to the RP faults region to form a (LaO) layer, which exists more stably under the octahedral crystal field [51,52]. This La ion migration process also makes the concentration of La elements in the RP faults region increase, which is higher than that in the bulk region. Therefore, annealing in a low vacuum produces an increase in oxygen vacancies, which will cause more La^2+^ to migrate to the RP faults region and stabilize to form (LaO), so that the white line ratio of the 90-0 film is higher than that of the 90-200 and 70-200 films. At the same time, the increase in oxygen vacancies leads to lattice expansion (or shrinkage) to produce strain, and the migration of La elements leads to local atomic ratio imbalance, which promotes the formation of RP faults. Therefore, the density of RP layer dislocations within the film under near-vacuum annealing is higher than that of the film under 200 T oxygen pressure annealing. This is also the reason that the RP fault density of the film deposited under 70 mT oxygen pressure is higher than that of the film deposited under 90 mT.

## 5. Conclusions

In summary, the LBSO films with three kinds of growing parameters were fabricated, with the films annealed in the oxygen-lacking growing environment displaying the increased density of faults and decreased light transmittance. Results of multiple modes in Cs-STEM including HAADF-STEM, EDS, and EELS revealed that such defects are mainly RP faults with local symmetry broken, different strain state, element distribution, and an electronic structure compared with that of the LBSO matrix. In particular, the quantitative EDS results showed that the La ion concentration in the RP faults region was doubled compared to that of the LBSO matrix. The EELS results showed that the M_5_/M_4_ value of the white line ratio of the enriched La ions at the RP faults region was increased compared to that of the bulk region, suggesting a change in the valence state of the La ions. It is noted that the oxygen deficiency also played a crucial role in regulating the above electronic structure, with the 90-0 LBSO film annealed under low oxygen partial pressure displaying the largest change of the white line ratio. These results suggested that oxygen deficiency reduced valence states from La^3+^ to La^2+^, which further migrated to the RP stacked region to enrich the formation of stable LaO structure. Meanwhile, the La migration led to local atomic disproportion and thereby the formation of more RP faults. This work reveals the modulation mechanism of light transmittance via oxygen deficiency bridged by the RP faults in LBSO films, one perovskite-typed TCO, which would provide theoretical guidance for the development of high-performance all-perovskite oxide devices.

## Figures and Tables

**Table 1:** The in-plane and out-of-plane lattice constants are calculated from the 103 RSM diagram.

	90 mT-200 T	70 mT-200 T	90 mT-0 T
out-of-plane	4.14 Å	4.14 Å	4.13 Å
in-plane	4.09 Å	4.09 Å	4.10 Å

## Data Availability

The original contributions presented in this study are included in the article. Further inquiries can be directed to the corresponding author.

## Supplementary Materials

The following supporting information can be downloaded at: https://www.mdpi.com/article/10.3390/ma18081696/s1, Figure S1. (a) HAADF image of the bulk region near the RP fault, and the yellow rectangular box is the quantitative EDS analysis area, (b) atomic proportion of the yellow rectangular region.; Figure S2. (a) HAADF image of the bulk region near the 1/2a<110> SFs, and the yellow rectangular box is the quantitative EDS analysis area, (b) atomic proportion of the yellow rectangular region. Figure S3. (a) The XRR curves of 90-200, 70-200, 90-0 films, (b) the Omega/2Theta scan curve of 90-0 film. Figure S4. (a) O-K edges of RP faults and bulk region, (b) Ba and La-M edges of bulk region.
