# Supplementary material for: Oxygen Deficiency Modulated La-Doped BaSnO3 Films Showing Improved Light Transmittance
Source: Materials (Basel). 2025 Apr 8;18(8):1696. doi: 10.3390/ma18081696 (PMC12028377; doi:10.3390/ma18081696)
Supplement: Supplementary file 1 [file materials-18-01696-s001.zip › materials-3520262-supplementary.pdf]

materials

MDPI

Supplementary Materials

Oxygen Deficiency Modulated La-Doped $BaSnO_3$ Films Showing Improved Light Transmittance

Kai Wu [1,2], Wan-Rong Geng [1,*], Yin-Lian Zhu [1,3] and Xiu-Liang Ma [1,2,4]

1 Bay Area Center for Electron Microscopy, Songshan Lake Materials Laboratory, Dongguan 523808, China; wukai@sslab.org.cn (K.W.); zhuyinlian@sslab.org.cn (Y.-L.Z.); xlma@sslab.org.cn (X.-L.M.)
2 Institute of Physics, Chinese Academy of Sciences, Beijing 100190, China
3 School of Materials Science and Engineering, Hunan University of Science and Technology, Xiangtan 411201, China
4 Quantum Science Center of Guangdong-Hong Kong-Macau Greater Bay Area, Shenzhen 518000, China
* Correspondence: gengwanrong@sslab.org.cn

Academic Editor: Giorgio Biasiol

Received: 23 February 2025
Revised: 2 April 2025
Accepted: 7 April 2025
Published: 8 April 2025

Citation: Wu, K.; Geng, W.-R.; Zhu, Y.-L.; Ma, X.-L. Oxygen Deficiency Modulated La-Doped $BaSnO_3$ Films Showing Improved Light Transmittance. *Materials* **2025**, *18*, 1696. https://doi.org/10.3390/ma18081696

Copyright: © 2025 by the authors. Licensee MDPI, Basel, Switzerland. This article is an open access article distributed under the terms and conditions of the Creative Commons Attribution (CC BY) license (https://creativecommons.org/licenses/by/4.0/).

1. Quantitative EDS analysis

(a)

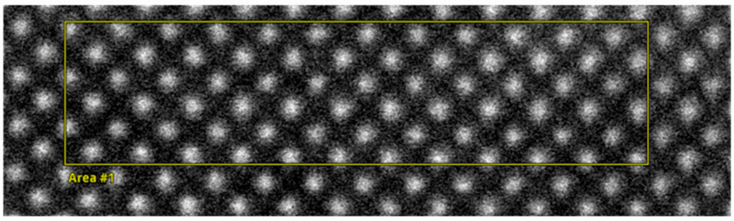

(b)

element	Sn	Ba	La
Atomic fraction (%)	43.71	53.68	2.6
Atomic Error (%)	2.94	3.04	0.33

Figure S1. (a) HAADF image of the bulk region near the RP fault, and the yellow rectangular box is the quantitative EDS analysis area, (b) atomic proportion of the yellow rectangular region.

Figure S1 shows the HAADF image of the perfect block region near the RP fault. Velox software is used to quantitatively analyze the atomic proportion in the yellow region, in which the element La accounts for 2.6% and the element Ba accounts for 53.6%. The ratio of La element to Ba element is 0.046, which is close to the doping ratio of 0.05. The ratio of La/Ba element is much lower than that of RP fault area, indicating that La element is enriched in RP fault area.

(a)

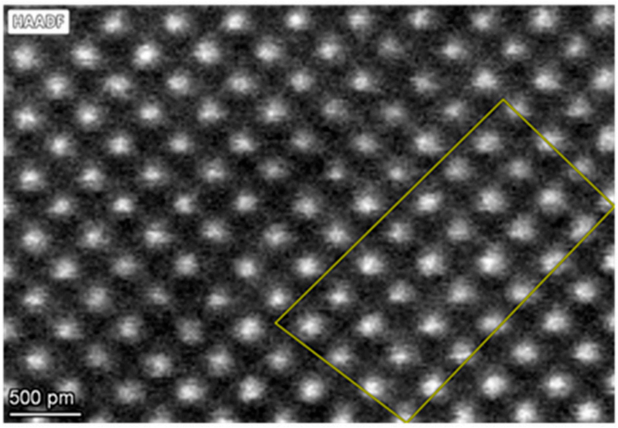

(b)

element	Sn	Ba	La
Atomic fraction (%)	48.30	49.13	2.57
Atomic Error (%)	2.94	3.04	0.35

Figure S2. (a) HAADF image of the bulk region near the 1/2a<110> SFs, and the yellow rectangular box is the quantitative EDS analysis area, (b) atomic proportion of the yellow rectangular region.

Figure S2 shows the HAADF image of the perfect block region near the 1/2a<110> SFs. Velox software is used to quantitatively analyze the atomic proportions in the yellow region, in which the proportion of La element is 2.57% and that of Ba element is 49.13%. The ratio of La element to Ba element is 0.049, which is close to the doping ratio of 0.05. La/Ba element ratio lower than that of 1/2a<110> SFs area.

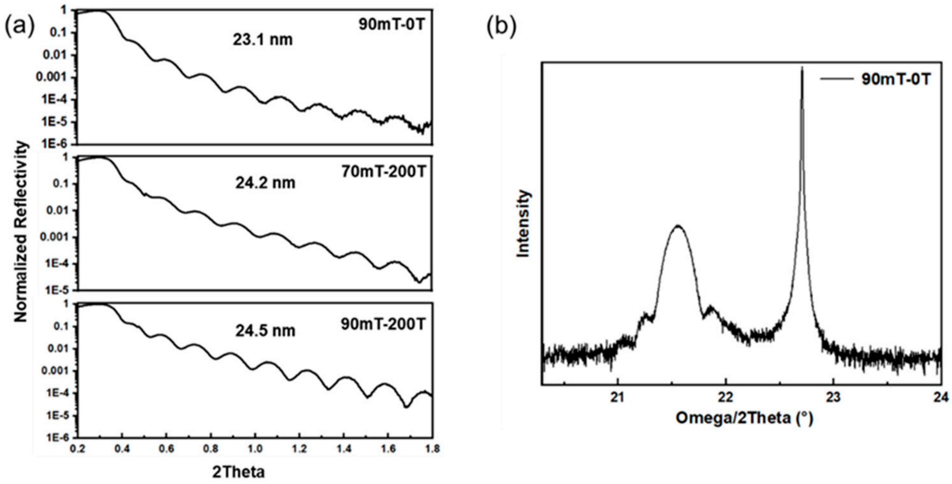

Figure S3. (a) The XRR curves of 90-200, 70-200, 90-0 films, (b) the Omega/2Theta scan curve of 90-0 film.

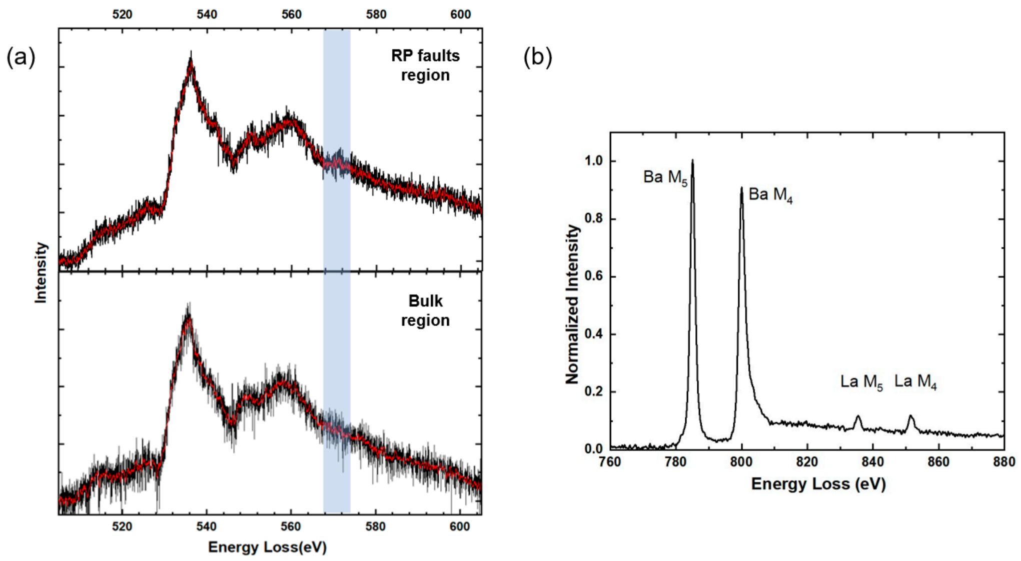

Figure S4. (a) O-K edges of RP faults and bulk region, (b) Ba and La-M edges of bulk region.
